# Personality traits explain levels of norm compliance during the COVID-19 pandemic

**DOI:** 10.3389/fpsyg.2026.1749198

**Published:** 2026-06-15

**Authors:** Oksana Zinchenko, Viktor Timokhov, Ekaterina Kosova, Vasily Klucharev, Todd A. Hare

**Affiliations:** 1 Centre for Cognition and Decision Making, Institute for Cognitive Neuroscience, National Research University Higher School of Economics, Moscow, Russia; 2 Department of Economics, Zurich Center for Neuroeconomics, University of Zurich, Zurich, Switzerland; 3 Faculty of Social Sciences, National Research University Higher School of Economics, Moscow, Russia

**Keywords:** cooperation, COVID-19, empathy, norm compliance, public goods game (PGG)

## Abstract

Social norms refer to unwritten rules or expectations that guide people's behavior in a given social setting. Norms often dictate how people should behave and communicate, among other things. Recent studies showed pronounced cross-cultural and individual differences in norm compliance. The current study explored the role of individual propensity to cooperate, culture and personality traits, such as Dark Triad and empathy (measured by Davis's questionnaire), in norm compliance to a newly introduced norm of wearing masks in public as a protective measure against COVID-19 in 2020–2021. In the current study, we investigated Swiss and Russian resident participants while they performed a public goods game (PGG) with additional reward and punishment conditions and completed the questionnaires on attitudes toward the norm of mask-wearing during COVID-19, interpersonal reactivity index and personality traits (narcissism, machiavellianism, and psychopathy). We found that individual motivation to cooperate or defect in a modified PGG did not predict the level of compliance to the protective norm of mask-wearing, while personality traits, such as Emphatic Concern, were significantly positively associated with the self-reported level of compliance with the norm of mask-wearing.

## Introduction

Social norms are the expectations that guide behavior in groups and societies crucial in maintaining social order, facilitating social interaction and promoting cooperation (Cialdini et al., 1991). However, numerous behavioral economics studies have identified so-called free-riders, individuals who sacrifice the group interest in favor of their own gain, in most groups (Camerer and Fehr, 2004). On average, about half of group members turn out to be “conditionally cooperative”—they monitor the other group members' behavior and comply with the norm only if they see that the other beneficiaries are willing to contribute to the group's welfare (Fischbacher et al., 2001). One way to promote cooperation would be to introduce a set of actions that group members can perform to prevent non-cooperative behavior from happening again, which is called social punishment (Fehr and Fischbacher, 2004). Fehr and Gächter (2000) showed that the opportunity for other members of a social group to punish those who deviate from the group standard (or those unwilling to contribute to the group gain) caused a large increase in cooperation.

Individual differences play an important role in norm compliance, including individual differences in working memory (Xie et al., 2020), prosocial orientation (Perugini and Gallucci, 2001), conscientiousness (Fiddick et al., 2016), and Big Five personality traits (Ludeke et al., 2021). Other factors that could influence norm compliance are country-level characteristics, such as the level of individualism (collectivism) and power distance in a country. Furthermore, a recent study suggested that such country-level characteristics can explain cross-cultural differences in decision-making better than individual differences (Anlló et al., 2024).

Descriptive social norms—individuals' perceptions of what others commonly do—are conceptually distinct from injunctive norms, which reflect perceived social approval, and have been shown to robustly shape health behavior during the COVID-19 pandemic, including causal increases in mask wearing (Heiman et al., 2023). In the current study, we addressed the role of individual differences, such as a personality traits and individual propensity to cooperate, together with country-level and/or demographic factors in compliance to the newly introduced social norm of mask-wearing in a period of the COVID-19 pandemic (December 2020–February 2021). In both Russia and Switzerland, mask-wearing was formally mandated by national or regional regulations during this period. In Russia, federal and regional decrees required mask use in indoor public spaces, though enforcement was uneven across regions. In Switzerland, the Federal Council introduced nationwide mask requirements in indoor public areas, following earlier cantonal-level mandates. Despite their legal status, compliance with these rules in both countries depended heavily on informal enforcement, peer behavior, and local attitudes. Thus, mask-wearing represented a protective behavior that was simultaneously shaped by formal legal mandates and descriptive social norms, making it well-suited for investigating the psychological and cultural determinants of norm compliance.

We hypothesized that the individual disposition to cooperate or betray group interests, which can be explored using behavioral economics paradigms, can effectively explain the level of compliance with a descriptive social norm, such as an obligation to wear masks in public places (Hypothesis I). We also hypothesized that personality traits, such as level of empathy and Dark Triad traits, can significantly influence the propensity to follow the social norm (Hypothesis II). To address these hypotheses, we collected data in two countries with the opposite individualism-collectivism and power distance scores, namely in Russian Federation (power distance = 93, individualism = 39) and Switzerland (power distance = 34, individualism = 68), and probed potential cross-cultural differences.

## Methods

### Procedure

In total, 135 participants took part in this study, including 66 Russian resident participants [42 female, mean age = 23.27 (±4.6)] and 69 participants residing in Switzerland [42 female, mean age = 23.32 (±2.7)]. All participants were at least 18 years old and had at least undergraduate education or were currently undergraduate students. The participants were recruited independently in each country. All the participants were either native Russian speakers (Russian sample) or spoke English fluently (Swiss sample). Before the experiment, the participants were given information about the study and were asked to sign consent forms. All experimental protocols were approved by National Research University Higher School of Economics Committee on Interuniversity Surveys and Ethical Assessment of Empirical Research in accordance with the Declaration of Helsinki. Informed consent was obtained from all subjects.

All participants were invited to join the online Zoom session where they were introduced to other group members. Each group consisted of four randomly assigned members, who previously had no social interactions with each other. After explaining the procedure of the online experiment, the investigator shared the link to the Gorilla.Sc platform to collect the demographic data and start the online public goods game (PGG). Table 1 provides a summary of demographic data. The online Zoom session ensured that participants believed in social nature of the PGG. After completing the game, participants were asked to fill out personality questionnaires at the same platform. After the experiment, all participants were contacted several days later to deliver a monetary outcome of the PGG and a participation fee.

**Table 1:** Summary of demographic data.

Summary of demographic data (without outliers)												
Feature	Russian sample						Swiss sample					
	Min	IQ	Median	Mean	3Q	Max	Min	IQ	Median	Mean	3Q	Max
Age	18	20	21	23.2	25.3	35	18	21	24	23.6	25	29
Number of roommates	1	2	3	3	4	8	1	3	3	3.4	4	8

See Data Preprocessing Section for more information about outliers.

Min, minimum; 1Q, first quartile; 3Q, third quartile; Max, maximum.

Each online PPG involved four players, although the responses of three players were prerecorded in a previous study by Sefton and colleagues (Sefton et al., 2007). Overall, participants played four different experimental conditions with the same group members: a (1) control, (2) reward, (3) punishment, and (4) combined reward and punishment condition. At the beginning of each round (10 rounds in total for each experimental condition), the participant was given six tokens, which she/he could allocate between their private account and the group fund. At the end of a round, the number of tokens in the group fund was multiplied by 1.5 and shared equally among all group members. In reward, punishment and combined reward and punishment experimental conditions, after distributing their six tokens, the participants were then given the chance to reward or punish other members in the group. In each trial, participants learned how many tokens other players had contributed to the group fund that round and received six extra tokens for sanctions. Any unused tokens went into their private account. The amount of the participant's monetary reward for the experiment was determined based on the overall result of the game. Each token accumulated on a private account was paid at the rate of 10 kopecks (Russian sample) or 10 cents (Swiss sample), while each token received from the group fund was paid at the rate of five kopecks (Russian sample) or five cents (Swiss sample).

To determine the propensity to cooperate and to follow the group norm, we extracted the variable “*Resp-prev_med*” defined as the mean difference between the number of tokens a participant contributed to the group fund in the current round and the median contribution of other players in the previous round. This index captures the participant's adjustment to group behavior: values near zero indicate conformity, positive values reflect contributions exceeding the group norm (cooperative generosity), and negative values reflect contributions below the group norm (self-interest or betrayal of group interests). Thus, *Resp-prev_med* provides a graded measure of conformity that also reveals whether the individual tends toward cooperative or selfish deviations from the group standard. In addition, we calculated sums of the invested tokens for each type of experimental conditions (control, reward, punishment, and reward and punishment condition) of the PGG.

### Personality questionnaires

At the end of the study, participants filled in two questionaries. The Interpersonal Reactivity Index (IRI) questionnaire (Davis, 1983; Karyagina and Kukhtova, 2016) tested the interpersonal differences, such as empathic concern (EC), empathic distress (ED), “fantasy scale” (FS) and perspective-taking (PT). The Dark Triad test (Jonason and Webster, 2010; Kornilova et al., 2015) tested the level of Narcissism, Machiavellism and Psychopathy.

We selected empathy and Dark Triad traits as predictors because they capture complementary motivational orientations directly relevant to compliance with social norms. Empathy, and especially the Empathic Concern component, reflects sensitivity to the welfare of others and has been consistently associated with prosocial and cooperative behavior (Eisenberg and Miller, 1987; Van Lange, 2008). Recent research also shows that empathy promotes compliance with COVID-19 protective measures, including mask-wearing and physical distancing (Pfattheicher et al., 2020). In contrast, the Dark Triad traits—narcissism, Machiavellianism, and psychopathy (Paulhus and Williams, 2002)—represent socially aversive dispositions characterized by self-interest, callousness, and manipulativeness. Prior work demonstrates that higher levels of Dark Triad traits predict reduced cooperation in public goods dilemmas and greater willingness to violate social norms (Bereczkei et al., 2010). Importantly, recent large-scale evidence shows that Dark Factor personality traits were negatively associated with compliance to COVID-19 preventive behaviors (Zettler et al., 2022). Taken together, these constructs allowed us to test both the prosocial and antisocial personality pathways that may influence norm compliance in the context of the pandemic.

### Questionnaire on the attitudes toward a norm of mask-wearing during COVID-19

To evaluate the behavioral patterns and cognitive attitudes related to COVID-19 preventative measures such as mask-wearing, we used surveys by Knotek et al. (2020). The questionnaires were adapted to an online format and also translated for Russian-speaking sample by the authors of this study. The questions were combined to determine four scales:

- *Behavior_rate*—the scale combined the questions addressing the frequency of norm-compliant behavior.
- *Under_pressure_rate*—the scale combined the questions addressing norm-compliant behavior under sanctions, that is, if the rule or sanction was imposed by authorities.
- *Emotion_rate*—the scale combined the questions addressing the emotional state of the participant with regard to coronavirus precautions and its violations.
- *Cognitive_rate*—the scale combined the questions addressing cognitive attitudes and beliefs of the participant regarding coronavirus precautions.

Additionally, the sum of scores over all four scales was computed (*total_rate*).

### Data preprocessing

Raw data was aggregated from Gorilla.sc online experimental platform (experiment builder; http://gorilla.sc) for 135 participants (66 for Russian sample, 69 for Swiss sample). Table 2 shows all variables used in the analysis: demographic variables (*Age, Roommates, Gender_q, Residency_q, Children_q, Education_num, Health_num, Long_Illnesses_q*, and *Area_q*); Dark Triad test (Narcissism, Psychopathy, and Machiavellianism); IRI test (PT, FS, EC, PD, and General empathy); PGG variables (*Pure_response, Reward_response, Punishment_response, Rew_pun_response, Total_response*, and *Resp-prev_med*); COVID-19 questionnaires (*Behavior_rate, Under_pressure_rate, Emotion_rate, Cognitive_rate*, and *total_rate*). Demographic data was quantized in one-hot encoding (as “one-of-K” or with dummy variables) scheme, except for *Education_num, Health_num, Age*, and *Roommates*. The categories for each demographic variable are shown in Table 3. For IRI and Dark Triad questionnaires, as well as for the COVID-19 tests all scales were computed. The PGG data included sums of the invested tokens for each condition, total sum of the responses for all trials, and the *Resp-prev_med* variable.

**Table 2:** List of dependent and independent variables that were used for regression models.

Variable	Definition
Independent variables (demography)	
*Age, roommates*	Age and number of roommates
*Gender_q, Residency_q, Children_q, Education_num, Health_num, Long_Illnesses_q*, and *Area_q*	Gender, residency, having children, education level, health state, having long standing illnesses, and type of area of living, respectively
Independent variables (IRI and Dark Triad tests)	
*Narcissism, Psychopathy*, and *Machiavellianism*	Traits measured by the Dark Triad test
*PT, FS, EC*, and *PD*	Perspective taking, fantasy, empathic concern, and personal distress subscales measured by the IRI test, respectively
*General empathy*	General empathy trait measured by the IRI test
Independent variables (PGG)	
*Pure_response, Reward_response, Punishment_response, Rew_pun_response*, and *Total_response*	Sum of the invested tokens for each condition and total sum of all invested tokens (*Total_response*)
*Resp-prev_med*	Mean difference of the amount of invested tokens to the group fund in the current round with the median of the amount of invested tokens to the group fund of other players in the previous round
Dependent variables (1 per model)	
*Behavior_rate*	Frequency of norm compliance
*Under_pressure_rate*	“I would comply with the norm if the authorities required it”
*Emotion_rate*	“I feel comfortable/discomfortable”
C*ognitive_rate*	“I believe that the coronavirus...”
*Total_rate*	Sum of scores from all scales

**Table 3:** Encoding for the demographic variables used in the multiple regression analysis.

Variable name	Interpretation
*Gender_q_1*	0, Male; 1, Female/Prefer not to say
*Residency_q_1*	0, Russian sample; 1, Swiss
*Children_q_1*	0, no children; 1, has children
*Education_num*	3, Completed secondary school; 4, University/College /technical or vocational school; 5, Incomplete university degree (current student); 6, University (first) degree; 8, Post-graduate degree (in Russian sample: 7 for Master's degree, 8 for PhD)
*Health_num*	1, Very Bad; 2, Bad; 3, Fair; 4, Good; 5, Very good
*Long_Illnesses_q_1*	0, No long-term illness/Don't know/Prefer not to say; 1, Has long-term illness
*Area_q_1*	0, Urban area/Prefer not to say; 1, Rural area

Some of the Education codes are absent since no one chose some of the answers. See preprocessing code on OSF for more details.

Four people were removed from analysis due to the missing values for the variable *Age*, as well as two participants who provided implausibly high numbers of roommates (i.e., 256,000 and 50). A participant is considered an outlier if he or she differed from the mean value by more than three standard deviations for at least one of the numerical features. Overall, seven more people were removed from the dataset and 122 participants were considered for the data analysis (56 for Russian sample, 66 for Swiss sample). All preprocessing was conducted with R and Python libraries *pandas* and *numpy*.

### Data analysis

Pearson correlation were computed for the numerical regressors before being fitted to the linear models. All numerical regressors and dependent variable population differences between Russian and Swiss resident samples were tested with *t*-tests and Multivariate Analysis of Variance (MANOVA) with Pillai's Trace as the test statistic and univariate ANOVA as a *post-hoc* tests. To estimate the effect of regressors on each dependent variable and their uncertainty, four Bayesian linear regressions were estimated for each of the five dependent variables. Model 1 included only *Resp-prev_med* as independent variable (see Table 2), Model 2 was based on IRI and Dark Triad tests, while Model 3 and Model 4 included demographic variables (*Age, Roommates, Gender_q, Residency_q, Children_q, Education_num, Health_num, Long_Illnesses_q*, and *Area_q*) in addition to the variables from Model 1 and Model 2, respectively. For each of the Bayesian linear regression models, we use four chains with 1,000 samples for warmup and 8,000 samples drawn after warmup, resulting in 32,000 samples drawn in total. Models were then compared pairwise with leave-one-out (LOO) cross-validation (Model 1 vs. Model 3 and Model 2 vs. Model 4). Posterior predictive checking was also conducted for each model (see plots on the OSF repository). All the analysis was done in R (version 4.2.2). For Bayesian linear regression brms library (version 2.21.0) was used (Bürkner, 2017). The No-U-Turn Sampler in Stan (Hoffman and Gelman, 2014) was used to draw samples, which is the default method in brms. The default, Gaussian non-informative priors were used in all models.

## Results

### Bivariate correlations between measures

**Figure 1:**
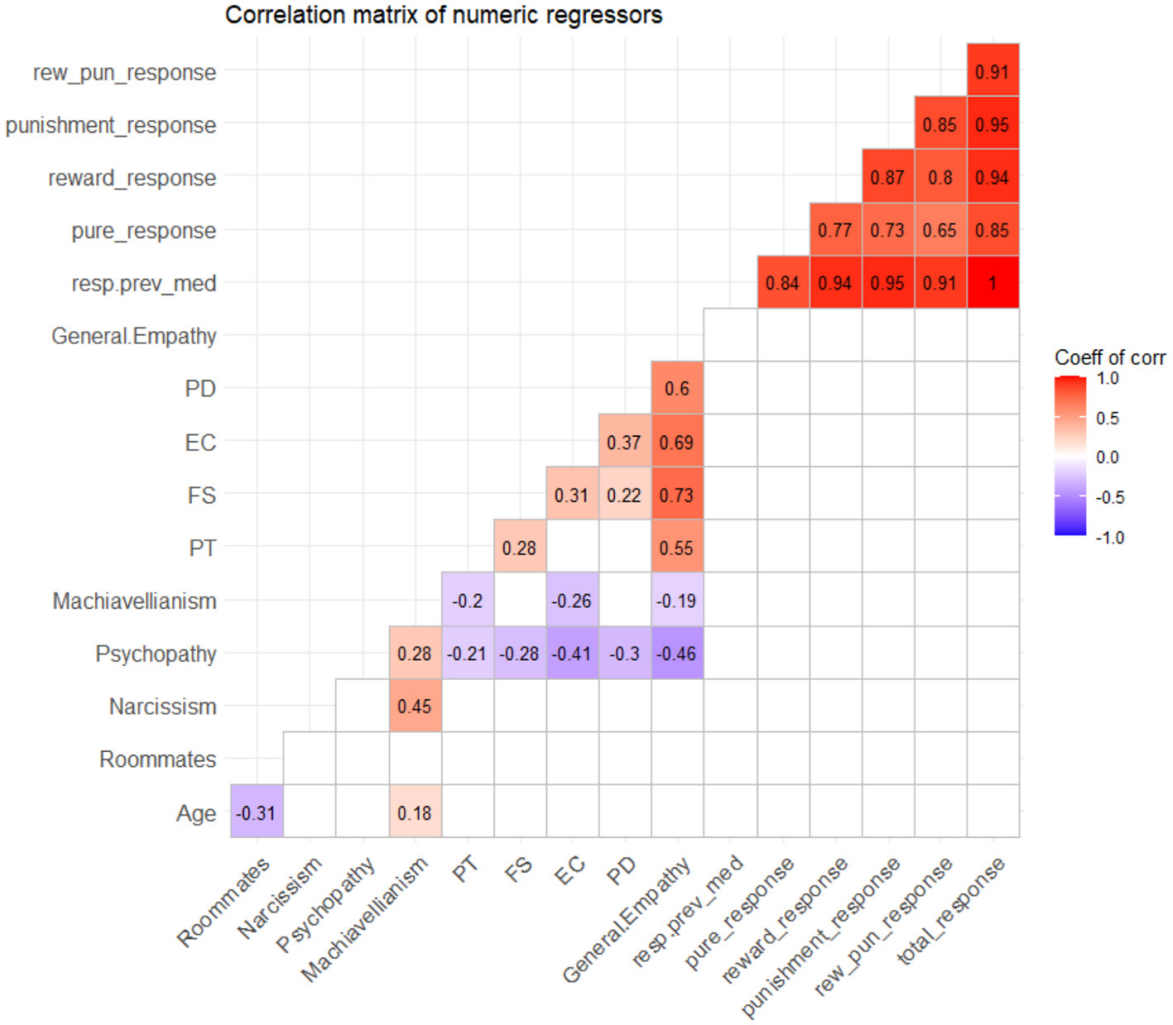
Correlation matrix for independent variables. During the preprocessing step *General empathy* and all game variables except *Resp.prev-med* were excluded from the further statistical analysis.

Figure 1 presents a correlation matrix for all numerical regressors. Only statistically significant correlations (*p*-value < 0.05) are plotted. Personality scales moderately correlate with each other, which can be explained by fuzzy boundaries between psychological constructs and shared latent factors (Figure 1). Thus, we removed *General empathy* variable from regression analysis, which is a sum of IRI subscales that we preserved.

PGG variables (*Resp-prev_med*; *Pure_response, Reward_response, Punishment_response, Rew_pun_response*, and *Total_response*, as in Table 3) were also highly correlated with each other. The highest correlation was found between *Resp-prev_med* and *Total_response* (*r* = 0.999) and the lowest between *Pure_response* and *Rew_pun_response* (*r* = 0.648). Therefore, we used only the *Resp.prev_med* variable to model the results of the PGG. *Resp.prev_med* is defined as the mean difference between a participant's contribution to the group fund in the current round and the median contribution of the other players in the previous round. This variable captures the extent to which participants adjusted their contributions to align with the group. A value close to zero indicates conformity to the group median, positive values indicate giving more than the group norm (reflecting cooperative generosity), and negative values indicate giving less (reflecting selfish or non-cooperative behavior).

**Figure 2:**
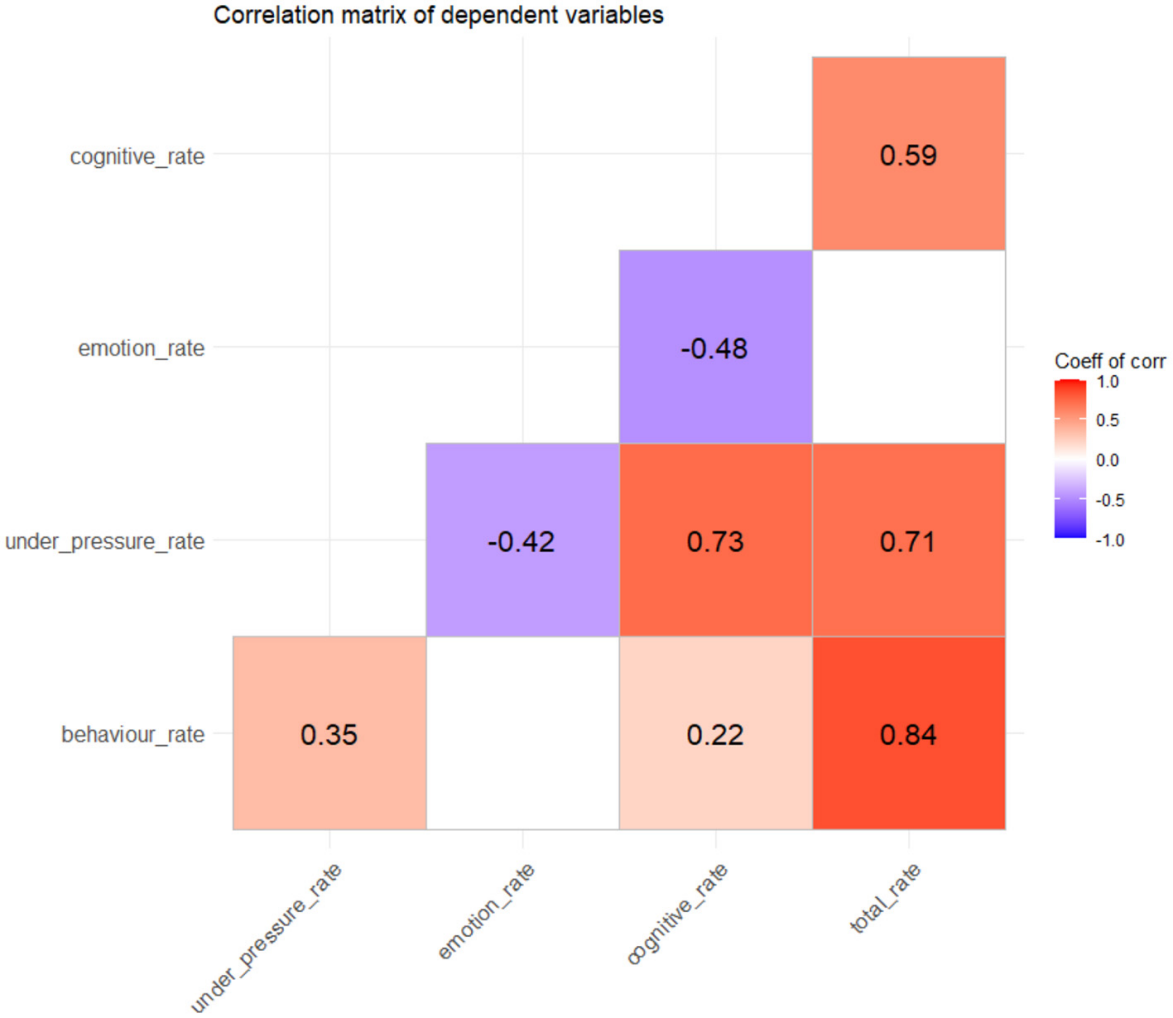
Correlation matrix for dependent variables. Bayesian regression analysis was performed on each of the dependent variables.

Although *total_rate* in social norm compliance questionnaire also correlated with three out of four subscales (see Figure 2), we still built separate models for subscales and the sum over them to distinguish possible effects of in-game behavior or personal traits on various aspects of social norm compliance.

### Cross-cultural differences

Intercultural differences between Swiss and Russian samples were estimated with *t*-tests for personality scales, variables collected in the PGG, and scales measuring COVID-19 behavioral patterns. Shapiro–Wilk test showed no significant difference from normal distribution for *Narcissism* (*W* = 0.98, *p* = 0.07), *PT* (*W* = 0.99, *p* = 0.32), *EC* (*W* = 0.98, *p* = 0.06), *PD* (*W* = 0.98, *p* = 0.07), *General empathy* (*W* = 0.98, *p* = 0.06), *Resp.prev_med* (*W* = 0.98, *p* = 0.06), *Punishment_response* (*W* = 0.98, *p* = 0.14), *Total_response* (*W* = 0.98, *p* = 0.08), *Behavior_rate* (*W* = 0.99, *p* = 0.27), and *Total_rate* (*W* = 0.98, *p* = 0.1). The following variables significantly differed from normal distribution: *Psychopathy* (*W* = 0.88, *p* < 0.001), *Machiavellianism* (*W* = 0.96, *p* = 0.002), *FS* (*W* = 0.97, *p* = 0.02), *Pure_response* (*W* = 0.97, *p* = 0.04), *Reward_response* (*W* = 0.98, *p* = 0.04), *Rew_pun_response* (*W* = 0.96, *p* = 0.002), *Under_pressure_rate* (*W* = 0.85, *p* < 0.001), *Emotion_rate* (*W* = 0.9, *p* < 0.001), and *Cognitive_rate* (*W* = 0.95, *p* < 0.001). Thus, the results of *t*-tests with non-normally distributed variables should be considered with caution.

Swiss and Russian samples did not significantly differ in Dark Triad traits [Pillai's Trace = 0.042, *F*_(3, 118)_ = 1.72, and *p* = 0.167]. Although MANOVA showed multivariate cross-cultural differences for the IRI [Pillai's Trace = 0.146, *F*_(4, 117)_ = 5.0, and *p* = 0.0009], none of the follow-up univariate ANOVAs reached significance threshold: PT [*F*_(1, 120)_ = 2.56, *p* = 0.112, *M*_ru_ = 26.2, *SD*_ru_ = 4.7, *M*_sw_ = 24.9, and *SD*_sw_ = 4.5], FS [*F*_(1, 120)_ = 2.61, *p* = 0.109, *M*_ru_ = 25.7, *SD*_ru_ = 5.2, *M*_sw_ = 24.2, and *SD*_sw_ = 5.1], EC [*F*_(1, 120)_ = 3.51, *p* = 0.063, *M*_ru_ = 25.5, *SD*_ru_ = 3.5, *M*_sw_ = 26.9, and *SD*_sw_ = 4.5], PD [*F*_(1, 120)_ = 3.20, *p* = 0.076, *M*_ru_ = 21.5, *SD*_ru_ = 4.7, *M*_sw_ = 20.0, and *SD*_sw_ = 4.6]. Furthermore, the mean *General empathy* also did not differ between samples [*t*_(118.92)_ = 1.38, *p* = 0.172, *M*_ru_ = 24.7, *SD*_ru_ = 2.9, *M*_sw_ = 24.0, and *SD*_sw_ = 3.09]. Therefore, the significant multivariate effect suggests that the groups differ in their empathy profiles. Specifically, the cultural variation might exist in the unique weighing and relationship between the IRI subscales rather than in a global empathy construct or any single dimension in isolation.

In the PGG, the Russian participants allocated more resources to the group fund than the Swiss participants both across all conditions [*t*_(111.09)_ = 3.93, *p* < 0.001, *M*_ru_ = 161.7, *SD*_ru_ = 46.0, *M*_sw_ = 130.5, and *SD*_sw_ = 40.9] and in each experimental conditions separately [Pillai's Trace = 0.142, *F*_(4, 117)_ = 4.82, and *p* = 0.001]: in a control [*F*_(1, 120)_ = 14.53, *p* = 0.0002, *M*_ru_ = 38.8, *SD*_ru_ = 10.9, *M*_sw_ = 31.7, and *SD*_sw_ = 9.8], reward [*F*_(1, 120)_ = 9.68, *p* = 0.002, *M*_ru_ = 41.8, *SD*_ru_ = 13.1, *M*_sw_ = 34.7, and *SD*_sw_ = 12.1], punishment [*F*_(1, 120)_ = 13.28, *p* = 0.0004, *M*_ru_ = 40.5, *SD*_ru_ = 12.8, *M*_sw_ = 32.3, and *SD*_sw_ = 12.1], and in combined reward and punishment experimental condition [*F*_(1, 120)_ = 14.99, *p* = 0.0002, *M*_ru_ = 40.6, *SD*_ru_ = 14.0, *M*_sw_ = 31.8, and *SD*_sw_ = 11.0]. Furthermore, Russian participants, in average, donated more than the group donated in the previous trial compared to Swiss participants (*resp-prev_med*): *t*_(110.99)_ = 3.97, *p* < 0.001, *M*_ru_ = 1.1, *SD*_ru_ = 1.2, *M*_sw_ = 0.3, *SD*_sw_ = 1.0. The *resp-prev_med* value in the Swiss group was closer to 0 indicate that Swiss participants tended to adjust the amount of invested tokens to the observed group behavior. Russian participants demonstrated more generous behavior than Swiss participants showing positive values of resp-prev. It suggests that in the current trial Russian participants on average contributed to the group fund more tokens than the group of a previous trial.

Finally, the two cultural groups reported similar mask-wearing rates during the COVID-19 pandemic, but this behavior was partially driven by different factors. The two samples did not differ in the total scores on the COVID-19 behavioral patterns test [*t*_(113.17)_ = −1.41, *p* = 0.16, *M*_ru_ = 258.2, *SD*_ru_ = 30.6, *M*_sw_ = 265.7, and *SD*_sw_ = 28.2], indicating that both Russian and Swiss resident samples expressed rather similar overall behavioral patterns during the COVID-19 pandemic. Specifically, there was no significant difference in the scores on *Behavior rate* [*t*_(109.03)_ = −0.16, *p* = 0.87, *M*_ru_ = 110.5, *SD*_ru_ = 21.2, *M*_sw_ = 111.1, and *SD*_sw_ = 18.2]. However, the Swiss resident participants scored higher than Russian resident participants on *Under pressure rate* [*t*_(103.99)_ = −3.39, *p* < 0.001, *M*_ru_ = 45.6, *SD*_ru_ = 11.9, *M*_sw_ = 52.3, and *SD*_sw_ = 9.4] and *Cognitive rate* [*t*_(113.77)_ = −3.2, *p* = 0.002, *M*_ru_ = 50.4, *SD*_ru_ = 9.2, *M*_sw_ = 55.6, and *SD*_sw_ = 8.6], while Swiss resident participants scored lower than Russian resident participants on *Emotion rate* [*t*_(105.47)_ = 3.08, *p* = 0.003, *M*_ru_ = 51.6, *SD*_ru_ = 9.6, *M*_sw_ = 46.7, and *SD*_sw_ = 7.8]. Thus, although the behavioral patterns were similar, the reasons for following norms differed.

### Bayesian linear regression

**Figure 3:**
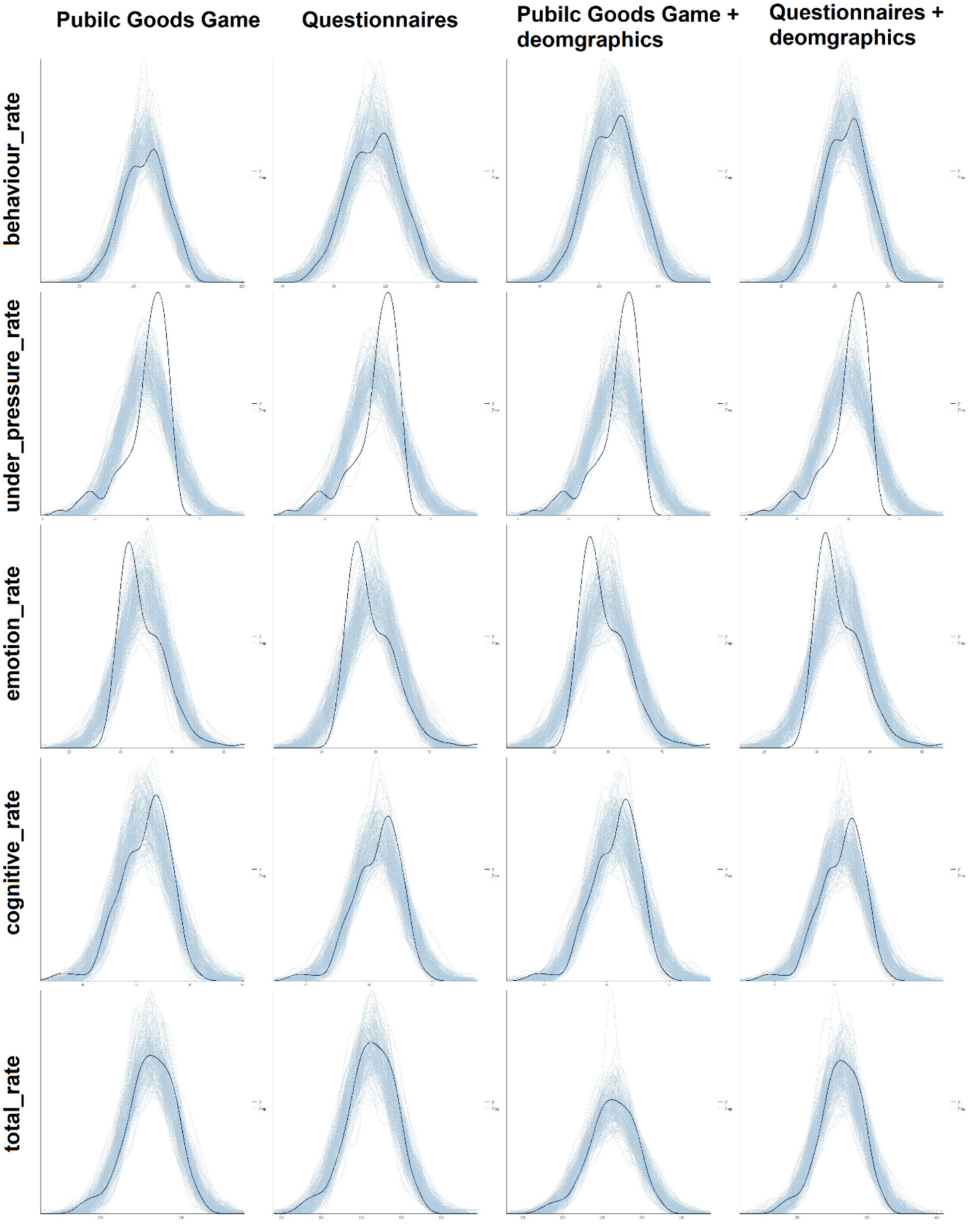
Posterior predictive checking plots. See code on the OSF repository for individual plots.

Table 3 provides interpretation of encoding demographic variables. Table 2 lists description of all independent and dependent variables used in the analysis. R-hat statistics were very close to 1.00 (< 1.01) for each parameter in each model indicating parameter convergence. The ratios of effective sample size to total posterior sample draws (*Neff_ratio*) also indicated good parameter convergence with values generally higher than 0.75 (see Tables 4–8). Posterior predictive checking plots did not show any implausible distributions (see Figure 3). If there is no null value in the 95% confidence interval of the beta parameter estimation, we consider it to differ from null and to be able to explain the dependent variable.

**Table 4:** Bayesian linear regression model for *Behavior_rate*.

Variable	LOO (elpd ±se)	Mean β ±se	95%-CI	*Neff_ratio*
**Model 1 (PGG)**	0 ± 0			
(Intercept)		110.46 ± 2.03	[106.46, 114.46]^*^	0.79
*resp.prev_med*		0.63 ± 1.54	[−2.42, 3.68]	0.77
**Model 3 (PGG** **+** **demographics)**	−4.2 ± 4.3			
(Intercept)		58.50 ± 21.69	[16.17, 101.49]^*^	0.86
*resp.prev_med*		1.43 ± 1.67	[−1.82, 4.71]	0.82
*Gender_q_1*		0.95 ± 3.93	[−6.78, 8.66]	0.80
*Residency_q_1*		2.33 ± 4.48	[−6.40, 11.03]	0.81
*Children_q_1*		−0.14 ± 5.94	[−11.72, 11.51]	0.80
*Long_Illnesses_q_1*		8.59 ± 4.86	[−0.90, 18.11]	0.79
*Area_q_1*		4.63 ± 5.54	[−6.17, 15.57]	0.81
*Age*		0.80 ± 0.65	[−0.47, 2.08]	0.81
*Roommates*		0.15 ± 1.29	[−2.35, 2.66]	0.82
*Education_num*		1.90 ± 1.36	[−0.77, 4.57]	0.80
*Health_num*		4.17 ± 3.74	[−3.18, 11.50]	0.82
**Model 2 (IRI** **+** **Dark Triad)**	0.0 ± 0.0			
(Intercept)		56.00 ± 20.38	[15.82, 96.47]^*^	0.86
Narcissism		−0.17 ± 0.55	[−1.26, 0.93]	0.81
Psychopathy		0.36 ± 0.69	[−0.99, 1.72]	0.78
Machiavellianism		1.07 ± 0.62	[−0.16, 2.29]	0.82
PT		0.30 ± 0.40	[−0.49, 1.09]	0.81
FS		−0.04 ± 0.37	[−0.78, 0.69]	0.83
EC		1.63 ± 0.50	[0.64, 2.62]^*^	0.84
PD		−0.25 ± 0.42	[−1.07, 0.57]	0.84
**Model 4 (IRI** **+** **Dark Triad** **+** **demographics)**	−6.1 ± 3.4			
(Intercept)		22.60 ± 30.06	[−36.71, 82.21]	0.84
Narcissism		−0.57 ± 0.59	[−1.74, 0.60]	0.80
Psychopathy		0.43 ± 0.73	[−0.99, 1.87]	0.78
Machiavellianism		1.04 ± 0.64	[−0.21, 2.28]	0.81
PT		0.29 ± 0.43	[−0.57, 1.14]	0.80
FS		−0.09 ± 0.40	[−0.87, 0.70]	0.82
EC		1.47 ± 0.55	[0.38, 2.54]^*^	0.80
PD		−0.13 ± 0.45	[−1.01, 0.75]	0.78
*Gender_q_1*		0.42 ± 3.97	[−7.42, 8.20]	0.81
*Residency_q_1*		−0.41 ± 4.56	[−9.38, 8.53]	0.80
*Children_q_1*		−2.56 ± 6.06	[−14.54, 9.41]	0.81
*Long_Illnesses_q_1*		8.65 ± 4.78	[−0.75, 18.11]	0.80
*Area_q_1*		4.85 ± 5.65	[−6.18, 15.93]	0.81
*Age*		0.65 ± 0.66	[−0.65, 1.94]	0.79
*Roommates*		0.46 ± 1.30	[−2.08, 3.04]	0.83
*Education_num*		1.58 ± 1.40	[−1.20, 4.34]	0.77
*Health_num*		3.19 ± 3.81	[−4.29, 10.74]	0.83

^*^Confidence interval does not include 0. Elpd, expected log pointwise predictive density (0 is the best value); Neff_ratio, recent Effective Sample size in a chain/total post-warmup draws (closer to 1 is the better).

**Table 5:** Bayesian linear regression model for *Under_pressure_rate*.

Variable	LOO (elpd ±se)	Mean β ±se	95%-CI	*Neff_ratio*
**Model 1 (PGG)**	−1.7 ± 4.2			
(Intercept)		49.41 ± 1.15	[47.14, 51.66]^*^	0.75
*resp.prev_med*		−0.14 ± 0.89	[−1.88, 1.59]	0.72
**Model 3 (PGG** **+** **Demographics)**	0.0 ± 0.0			
(Intercept)		39.02 ± 11.67	[16.07, 61.59]^*^	0.87
*resp.prev_med*		1.05 ± 0.92	[−0.76, 2.86]	0.80
*Gender_q_1*		2.92 ± 2.13	[−1.26, 7.10]	0.78
*Residency_q_1*		7.86 ± 2.45	[3.10, 12.70]^*^	0.81
*Children_q_1*		−3.36 ± 3.22	[−9.74, 2.94]	0.82
*Long_Illnesses_q_1*		5.05 ± 2.59	[−0.03, 10.13]	0.79
*Area_q_1*		1.19 ± 3.03	[−4.80, 7.12]	0.82
*Age*		−0.27 ± 0.35	[−0.96, 0.42]	0.84
*Roommates*		0.49 ± 0.70	[−0.88, 1.88]	0.80
*Education_num*		0.57 ± 0.73	[−0.88, 2.01]	0.81
*Health_num*		1.10 ± 2.04	[−2.96, 5.10]	0.79
**Model 2 (IRI** **+** **Dark Triad)**	−1.9 ± 4.5			
(Intercept)		34.28 ± 11.42	[11.79, 56.68]^*^	0.84
Narcissism		0.13 ± 0.31	[−0.48, 0.73]	0.81
Psychopathy		−0.44 ± 0.38	[−1.19, 0.31]	0.81
Machiavellianism		−0.19 ± 0.35	[−0.87, 0.49]	0.81
PT		0.04 ± 0.23	[−0.41, 0.50]	0.80
FS		0.23 ± 0.21	[−0.19, 0.65]	0.82
EC		0.55 ± 0.29	[−0.02, 1.10]	0.84
PD		−0.13 ± 0.24	[−0.59, 0.34]	0.80
**Model 4 (IRI** **+** **Dark Triad** **+** **demographics)**	0.0 ± 0.0			
(Intercept)		23.47 ± 15.95	[−7.68, 54.70]	0.88
Narcissism		0.08 ± 0.32	[−0.54, 0.70]	0.76
Psychopathy		−0.62 ± 0.39	[−1.39, 0.14]	0.78
Machiavellianism		0.09 ± 0.34	[−0.59, 0.75]	0.80
PT		0.27 ± 0.23	[−0.18, 0.73]	0.80
FS		0.31 ± 0.21	[−0.11, 0.73]	0.82
EC		0.19 ± 0.30	[−0.39, 0.77]	0.82
PD		0.05 ± 0.24	[−0.41, 0.51]	0.81
*Gender_q_1*		2.74 ± 2.13	[−1.45, 6.91]	0.80
*Residency_q_1*		8.23 ± 2.40	[3.49, 12.96]^*^	0.82
*Children_q_1*		−2.50 ± 3.22	[−8.84, 3.79]	0.83
*Long_Illnesses_q_1*		4.81 ± 2.58	[−0.24, 9.86]	0.78
*Area_q_1*		0.34 ± 2.99	[−5.56, 6.20]	0.80
*Age*		−0.26 ± 0.35	[−0.95, 0.42]	0.79
*Roommates*		0.64 ± 0.69	[−0.73, 2.00]	0.81
*Education_num*		0.18 ± 0.75	[−1.28, 1.64]	0.83
*Health_num*		0.96 ± 2.03	[2.98, 4.90]	0.83

^*^Confidence interval does not include 0. Elpd, expected log pointwise predictive density (0 is the best value); Neff_ratio, recent Effective Sample size in a chain / total post-warmup draws (closer to 1 is the better).

**Table 6:** Bayesian linear regression model for *Emotion_rate*.

Variable	LOO (elpd ±se)	Mean β ±se	95%-CI	*Neff_ratio*
**Model 1 (PGG)**	0.0 ± 0.0			
(Intercept)		49.03 ± 0.94	[47.20, 50.87]^*^	0.71
*resp.prev_med*		−0.25 ± 0.72	[−1.66, 1.16]	0.74
**Model 3 (PGG** **+** **demographics)**	−0.3 ± 4.5			
(Intercept)		61.99 ± 9.57	[43.34, 80.79]^*^	0.84
*resp.prev_med*		−1.01 ± 0.74	[−2.45, 0.44]	0.80
*Gender_q_1*		−2.17 ± 1.74	[−5.58, 1.24]	0.81
*Residency_q_1*		−4.45 ± 1.98	[−8.34, −0.58]^*^	0.80
*Children_q_1*		3.50 ± 2.60	[−1.68, 8.59]	0.78
*Long_Illnesses_q_1*		−2.39 ± 2.13	[−6.58, 1.77]	0.83
*Area_q_1*		−0.34 ± 2.45	[−5.17, 4.47]	0.78
*Age*		−0.01 ± 0.28	[−0.57, 0.55]	0.83
*Roommates*		0.01 ± 0.56	[−1.10, 1.12]	0.83
*Education_num*		0.53 ± 0.59	[−0.64, 1.68]	0.83
*Health_num*		−2.75 ± 1.66	[−6.01, 0.52]	0.83
**Model 2 (IRI** **+** **Dark Triad)**	0.0 ± 0.0			
(Intercept)		42.97 ± 9.66	[24.00, 61.82]^*^	0.84
Narcissism		0.16 ± 0.26	[−0.35, 0.67]	0.82
Psychopathy		0.12 ± 0.33	[−0.51, 0.77]	0.80
Machiavellianism		0.19 ± 0.29	[−0.38, 0.77]	0.84
PT		−0.02 ± 0.19	[−0.40, 0.36]	0.82
FS		−0.08 ± 0.18	[−0.43, 0.28]	0.79
EC		−0.04 ± 0.24	[−0.51, 0.43]	0.82
PD		0.23 ± 0.20	[−0.16, 0.62]	0.79
**Model 4 (IRI** **+** **Dark Triad** **+** **demographics)**	−1.0 ± 4.1			
(Intercept)		56.23 ± 13.64	[29.24, 82.78]^*^	0.81
Narcissism		0.14 ± 0.27	[−0.39, 0.67]	0.78
Psychopathy		0.23 ± 0.33	[−0.41, 0.88]	0.80
Machiavellianism		0.05 ± 0.29	[−0.54, 0.62]	0.81
PT		−0.14 ± 0.20	[−0.54, 0.25]	0.80
FS		−0.14 ± 0.18	[−0.50, 0.22]	0.78
EC		0.19 ± 0.25	[−0.30, 0.69]	0.81
PD		0.11 ± 0.20	[−0.28, 0.51]	0.81
*Gender_q_1*		−2.04 ± 1.85	[−5.66, 1.58]	0.79
*Residency_q_1*		−4.37 ± 2.07	[−8.41, −0.27]^*^	0.81
*Children_q_1*		3.53 ± 2.76	[−1.91, 8.93]	0.82
*Long_Illnesses_q_1*		−2.85 ± 2.19	[−7.15, 1.47]	0.79
*Area_q_1*		−0.18 ± 2.54	[−5.15, 4.84]	0.81
*Age*		0.06 ± 0.30	[−0.53, 0.64]	0.80
*Roommates*		0.07 ± 0.58	[−1.07, 1.20]	0.80
*Education_num*		0.49 ± 0.64	[−0.75, 1.76]	0.78
*Health_num*		−2.85 ± 1.74	[−6.27, 0.59]	0.80

^*^Confidence interval does not include 0. Elpd, expected log pointwise predictive density (0 is the best value); Neff_ratio, recent Effective Sample size in a chain / total post-warmup draws (closer to 1 is the better).

**Table 7:** Bayesian linear regression model for *Cognitive_rate*.

Variable	LOO (elpd ±se)	Mean β ±se	95%-CI	Neff_ratio
**Model 1 (PGG)**	−1.8 ± 4.7			
(Intercept)		53.32 ± 0.97	[51.40, 55.22]^*^	0.76
*resp.prev_med*		−0.11 ± 0.74	[−1.54, 1.34]	0.76
**Model 3 (PGG** **+** **demographics)**	0.0 ± 0.0			
(Intercept)		40.28 ± 9.82	[20.98, 59.37]^*^	0.80
resp.prev_med		0.98 ± 0.76	[−0.49, 2.48]	0.82
*Gender_q_1*		3.26 ± 1.79	[−0.31, 6.77]	0.77
*Residency_q_1*		5.60 ± 2.02	[1.62, 9.60]^*^	0.80
*Children_q_1*		−1.75 ± 2.68	[−6.98, 3.47]	0.82
*Long_Illnesses_q_1*		3.40 ± 2.18	[−0.84, 7.69]	0.80
*Area_q_1*		1.54 ± 2.50	[−3.39, 6.45]	0.81
*Age*		−0.27 ± 0.29	[−0.84, 0.30]	0.79
*Roommates*		0.27 ± 0.58	[−0.88, 1.41]	0.78
*Education_num*		0.99 ± 0.61	[−0.21, 2.19]	0.77
*Health_num*		1.68 ± 1.69	[−1.64, 5.02]	0.82
**Model 2 (IRI** **+** **Dark Triad)**	0.0 ± 0.0			
(Intercept)		49.04 ± 9.65	[30.19, 68.17]^*^	0.88
Narcissism		0.10 ± 0.26	[−0.42, 0.62]	0.84
Psychopathy		−0.32 ± 0.32	[−0.96, 0.31]	0.78
Machiavellianism		−0.29 ± 0.30	[−0.87, 0.29]	0.82
PT		−0.20 ± 0.19	[−0.58, 0.18]	0.75
FS		0.22 ± 0.18	[−0.13, 0.58]	0.78
EC		0.38 ± 0.24	[−0.09, 0.85]	0.81
PD		−0.13 ± 0.20	[−0.52, 0.26]	0.83
**Model 4 (IRI** **+** **Dark Triad** **+** **demographics)**	−0.2 ± 3.9			
(Intercept)		37.12 ± 13.75	[10.34, 64.12]^*^	0.82
Narcissism		0.03 ± 0.27	[−0.51, 0.55]	0.82
Psychopathy		−0.50 ± 0.33	[−1.16, 0.15]	0.81
Machiavellianism		−0.08 ± 0.29	[−0.65, 0.49]	0.79
PT		−0.01 ± 0.20	[−0.40, 0.38]	0.80
FS		0.26 ± 0.18	[−0.09, 0.62]	0.79
EC		0.06 ± 0.25	[−0.44, 0.55]	0.80
PD		0.03 ± 0.21	[−0.38, 0.43]	0.77
*Gender_q_1*		2.87 ± 1.83	[−0.71, 6.48]	0.81
*Residency_q_1*		5.51 ± 2.05	[1.44, 9.56]^*^	0.79
*Children_q_1*		−0.90 ± 2.76	[−6.38, 4.55]	0.80
*Long_Illnesses_q_1*		3.51 ± 2.19	[−0.80, 7.85]	0.83
*Area_q_1*		1.29 ± 2.54	[−3.68, 6.27]	0.81
*Age*		−0.21 ± 0.30	[−0.81, 0.37]	0.78
*Roommates*		0.27 ± 0.59	[−0.89, 1.43]	0.80
*Education_num*		0.64 ± 0.63	[−0.59, 1.89]	0.82
*Health_num*		1.72 ± 1.74	[−1.71, 5.10]	0.82

^*^Confidence interval does not include 0. Elpd, expected log pointwise predictive density (0 is the best value); Neff_ratio, recent Effective Sample size in a chain / total post-warmup draws (closer to 1 is the better).

**Table 8:** Bayesian linear regression model for *total_rate*.

Variable	LOO (elpd ±se)	Mean β ±se	95%-CI	*Neff_ratio*
**Model 1 (PGG)**	0.0 ± 0.0			
(Intercept)		262.20 ± 3.10	[256.10, 268.27]^*^	0.76
*Resp.prev_med*		0.14 ± 2.36	[-4.47, 4.77]	0.74
**Model 3 (PGG** **+** **demographics)**	−2.0 ± 4.2			
(Intercept)		199.33 ± 32.25	[136.04, 262.54]^*^	0.85
*resp.prev_med*		2.44 ± 2.49	[−2.42, 7.35]	0.75
*Gender_q_1*		4.91 ± 5.85	[−6.57, 16.37]	0.79
*Residency_q_1*		11.27 ± 6.62	[−1.73, 24.25]	0.80
*Children_q_1*		−1.76 ± 8.90	[−19.31, 15.56]	0.81
*Long_Illnesses_q_1*		14.58 ± 7.09	[0.67, 28.49]^*^	0.81
*Area_q_1*		7.11 ± 8.30	[−9.22, 23.35]	0.81
*Age*		0.27 ± 0.96	[−1.61, 2.14]	0.87
*Roommates*		0.93 ± 1.90	[−2.79, 4.68]	0.81
*Education_num*		3.98 ± 2.00	[0.09, 7.87]^*^	0.84
*Health_num*		4.22 ± 5.62	[−6.85, 15.23]	0.82
**Model 2 (IRI** **+** **Dark Triad)**	0.0 ± 0.0			
(Intercept)		182.06 ± 30.37	[122.27, 241.67]^*^	0.83
Narcissism		0.23 ± 0.82	[−1.39, 1.82]	0.82
Psychopathy		−0.27 ± 1.01	[−2.25, 1.72]	0.78
Machiavellianism		0.78 ± 0.91	[−1.02, 2.56]	0.79
PT		0.12 ± 0.61	[−1.07, 1.32]	0.79
FS		0.34 ± 0.56	[−0.76, 1.43]	0.81
EC		2.52 ± 0.75	[1.04, 4.00]^*^	0.77
PD		−0.27 ± 0.62	[−1.48, 0.93]	0.78
**Model 4 (IRI** **+** **Dark Triad** **+** **demographics)**	−5.0 ± 3.7			
(Intercept)		139.68 ± 44.46	[51.93, 227.43]^*^	0.85
Narcissism		−0.31 ± 0.86	[−2.01, 1.36]	0.83
Psychopathy		−0.48 ± 1.08	[−2.61, 1.64]	0.81
Machiavellianism		1.10 ± 0.94	[−0.74, 2.96]	0.82
PT		0.41 ± 0.65	[−0.86, 1.68]	0.83
FS		0.34 ± 0.59	[−0.81, 1.50]	0.81
EC		1.89 ± 0.81	[0.29, 3.48]^*^	0.80
PD		0.07 ± 0.65	[−1.21, 1.37]	0.80
*Gender_q_1*		4.07 ± 5.93	[−7.72, 15.60]	0.79
*Residency_q_1*		8.97 ± 6.67	[−4.12, 22.12]	0.77
*Children_q_1*		−2.33 ± 8.93	[−19.93, 15.31]	0.83
*Long_Illnesses_q_1*		14.14 ± 7.09	[0.14, 27.91]^*^	0.82
*Area_q_1*		6.28 ± 8.31	[−10.05, 22.62]	0.80
*Age*		0.23 ± 0.97	[−1.68, 2.14]	0.82
*Roommates*		1.42 ± 1.92	[−2.35, 5.18]	0.84
*Education_num*		2.89 ± 2.04	[−1.06, 6.91]	0.83
*Health_num*		3.01 ± 5.61	[−8.17, 14.03]	0.82

^*^Confidence interval does not include 0. Elpd, expected log point-wise predictive density (0 is the best value); Neff_ratio, recent Effective Sample size in a chain/total post-warmup draws (closer to 1 is the better).

### Frequency of norm compliance and emotional comfort—*behavior_rate* and *emotion_rate*

Neither *behavior_rate* nor *emotion_rate* could be predicted with PGG variables (see Tables 4, 6, respectively). When accounting for demographics, only *Residency_q_1* turned out to influence *emotion_rate* in both PGG [95%-CI of beta coefficient (−8.34, −0.58), mean ± se = −4.45 ± 1.98] and questionnaire [95%-CI of beta coefficient (−8.41, −0.27), mean ± se = −4.37 ± 2.07] models. These results are in line with *t*-tests results above, indicating higher *emotional_rate* in the Russian sample and a similar *behavior_rate* in the two samples. None of the model pairs showed any difference in the LOO criterion between models with and without demographics. Overall, Hypothesis I was rejected for both of these subscales.

None of the studied personality traits could predict *emotion_rate* both before and after accounting for demographics (see Table 6). Only *Residency_q_1* indicated a difference between Swiss and Russian samples [95%-CI of beta coefficient (−8.41, −0.27), mean ± se = −4.37 ± 2.07]. Nonetheless, increase in one of the IRI subscales, Empathic Concern, resulted in increase of *behavior_rate* [95%-CI of beta coefficient (0.64, 2.62), mean ± se = 1.63 ± 0.50; see Table 4]. This effect was preserved after accounting for demographics [95%-CI of beta coefficient (0.38, 2.54), mean ± se = 1.47 ± 0.55], although LOO criterion showed stronger evidence for the model without demographics. All other variables included null values in their confidence intervals. Thus, Hypothesis II was confirmed only for Empathic Concern in relation to *behavior_rate*.

### Compliance to enforced norms and cognitive beliefs about COVID-19—*under_pressure_rate* and *cognitive_rate*

Neither mask wearing norms that are enforced by the authority (*under_pressure_rate*) nor by cognitive beliefs about COVID-19 (*cognitive_rate*) could be explained by PGG variables (see Tables 5, 7). Accounting for demographics only replicated the difference shown above between Swiss and Russian samples via *Residency_q_1* variable for both *under_pressure_rate* [95%-CI of beta coefficient (3.10, 12.70), mean ± se = 7.86 ± 2.45] and *cognitive_rate* [95%-CI of beta coefficient (1.62, 9.60), mean ± se = 5.60 ± 2.02]. Thus, Hypothesis I was rejected for both subscales.

The models with Dark Triad and IRI variables could not explain *under_pressure_rate* and *cognitive_rate* (see Tables 5, 7). Again, accounting for demographics replicated the difference between samples for *under_pressure_rate* [95%-CI of beta coefficient (3.49, 12.96), mean ± se = 8.23 ± 2.40] and *cognitive_rate* [95%-CI of beta coefficient (1.44, 9.56), mean ± se = 5.51 ± 2.05]. However, Hypothesis II was rejected for both subscales.

### Overall index of norm compliance—*total_rate*

The index of social norm compliance during COVID-19, *total_rate*, is the sum of all four subscales, that is, *behavior_rate, emotion_rate, under_pressure_rate*, and *cognitive_rate*. PGG variable did not explain *total_rate* neither before accounting for demographics, not after (see Table 8). Higher level of education [95%-CI of beta coefficient (0.09, 7.87), mean ± se = 3.98 ± 2.00] and the presence of long-term illness [95%-CI of beta coefficient (0.67, 28.49), mean ± se = 14.58 ± 7.09] increased *total_rate*, but the uncertainty in parameter estimations is high indicating an inability of the model to determine the precise magnitude of effects with the current data. Model comparisons using the LOO criterion did indicate that models with and without demographics were equivalent. All in all, Hypothesis I was rejected for total index of norm compliance.

Among personality traits, only Empathic Concern (EC) [95%-CI of beta coefficient (1.04, 4.00), mean ± se = 2.52 ± 0.75] could explain *total_rate*. Accounting for demographics slightly decreased parameter estimation for EC [95%-CI of beta coefficient (0.29, 3.48), mean ± se = 1.89 ± 0.81], but it was non-zero. Among demographics, only the presence of long-term illness increased norm compliance, though the uncertainty in the parameter estimation was high [95%-CI of beta coefficient (0.14, 27.91), mean ± se = 14.14 ± 7.09]. However, with LOO criterion the model without demographics is preferred and better explains the data. To sum up, Hypothesis II was confirmed only for Empathic Concern in relation to the total index of norm compliance.

We hypothesized that the individual disposition to cooperate or betray group interests, which can be explored using behavioral economics paradigms, can effectively explain the level of compliance with a descriptive social norm, such as an obligation to wear masks in public places (Hypothesis I). We also hypothesized that personality traits, such as level of empathy and Dark Triad traits, can significantly influence the propensity to follow the social norm (Hypothesis II). Taken together, we did not find any evidence to support Hypothesis I. As for the Hypothesis II, only Empathic Concern (EC) subscale of IRI questionnaire could explain the overall index of social norm compliance (*total_rate*), as well as the frequency of norm compliance (*behavior_rate*). Thus, Hypothesis II was only partly supported.

## Discussion

In the current study, we explored the impact of individual differences, such as a personality traits and propensity to cooperate during the economic game, on norm-compliance behavior and on reported real-life mask-wearing behavior during COVID-19 restrictions. It is important to note that while mask use was legally mandated in both Russia and Switzerland at the time of data collection, actual adherence depended not only on awareness of the regulations but also on the informal social norms of one's environment. This dual nature—being both a formal requirement and a socially enforced behavior—makes mask-wearing an especially informative case for studying norm compliance during the pandemic. We found that propensity to cooperate measured in the PGG did not predict the strength of the cognitive beliefs toward COVID-19 regulations on mask-wearing (*cognitive_rate*) and the total level of self-reported compliance (*total_rate*). We found no such effect of cooperative tendencies during the public goods on all measures, including the emotional state of the participant regarding coronavirus precautions and its violations (*under_pressure_rate*), the self-reported propensity to follow the norm of mask-wearing (*behavioral_rate)* and the level of self-reported arousal/anxiety related to the norm of mask-wearing (*emotion_rate*). Therefore, our Hypothesis I has not been not supported. However, our results suggest that personality traits significantly affect the level of norm compliance with a norm of mask-wearing: we found that behavioral attitudes toward COVID-19 precautions and self-reported total level of the self-reported propensity to follow the norm of mask-wearing were positively associated with emphatic concern score measured by the Interpersonal Reactivity Index questionnaire, that partly support our initial Hypothesis II.

Our findings support the growing evidence of the importance of empathy in maintaining cooperation and norm-compliance. Low to moderate positive relations were found between empathy and both prosocial behavior and cooperative/socially competent behavior (Eisenberg and Miller, 1987). For more than a decade empathy was recognized as a key factor contributing to the display of prosocial behavior (Batson et al., 1991; Eisenberg, 2000). Our results are also in line with previous studies showing the association between empathy and prosocial behavior during the COVID-19 pandemic. Mellacher (2020) focused on changes in intragroup cooperation and covered not only the COVID-19 era, but also the pre-pandemic period (2019–2020). The results showed a rather high volatility of cooperativity in all time periods. Interestingly, during the pre-pandemic period, 67%−76% of the participants in the experiment were cooperative. In the first wave of the epidemic, immediately after the lockdown in Austria (where the study was conducted), the number of cooperators was 71% (i.e., within the pre-crisis norm), but by the second wave and the second lockdown the number of cooperators dropped dramatically to only 43%. However, the validity of this comparison may be questioned, since different people participated in the public good game at different periods of time. Therefore, we cannot trace changes in the propensity to cooperate under the influence of COVID-19 within individuals.

van de Groep et al. (2020) aimed to examine the effect of COVID-19 on individual changes in empathy and prosocial behavior in adolescents aged 10–20. Longitudinal studies showed that adolescents in general reacted differently to the epidemic crisis than the older members of society—stress and anxiety levels decreased in adolescents and, on the contrary, increased in adults (Kwong et al., 2021). The results of their study showed that during the pandemic, subjects demonstrated a decreased level of empathic concern as compared to their pre-pandemic levels, that can be explained by limited social interactions providing small opportunities for a prosocial activity. In addition, participants showed an increase in donations in the experimental condition (Kwong et al., 2021) to a friend (about 51% of the total), a doctor (78%), and to people with a weak immune system or COVID-19 (63% and 69%, respectively), while the level of donations to a stranger was relatively low (39%). The decrease in empathic concern, however, was most likely due to the forced restriction of social interaction during the lockdown. In our study data were collected after the lockdown, when access to the public places and a number of social interactions increased, so the level of empathic concern could also increase.

Recent studies also showed some indirect evidence suggesting the presence of conditionally cooperative behavior during the COVID-19 pandemic. Hagger et al. (2020) showed that subjective norm, moral norm, and predicted behavioral control were the key attributes of social distancing norm compliance for the Australian sample. This suggests that when deciding to comply with the norm of distancing, participants were not only guided by their internal attitudes but also by the behavior of their peers, which may indicate conditional cooperation. However, another cross-cultural study by Romano et al. (2021) that sought to examine trends in cooperation and trust failed to reveal a pattern between the overall level of cooperation in society and the propensity for more prosocial behavior in the COVID-19 era, which suggests against conditional conformity. Nevertheless, our study partially filled this gap to explore the impact of propensity to cooperate and individual differences on norm compliance with respect to COVID-19 norms and showed greater role of personality traits in norm compliance behavior and group factor (residency) when participants are presented with descriptive norms (in a case of a norm of mask-wearing).

## Conclusion

In our study, propensity to cooperate, measured in the PGG, did not predict the reported level of compliance with COVID-19 restrictions. We showed that emphatic concern as a personality trait is positively associated with the self-reported propensity to follow the norm of mask-wearing (*behavior_rate*) and the total level of norm compliance with respect to COVID-19 protective measures (*total_rate*).

## Data Availability

The datasets presented in this study can be found in online repositories. The names of the repository/repositories and accession number(s) can be found below: https://osf.io/2pwgt/.
